# Beneficial Effects of Tianeptine on Hippocampus-Dependent Long-Term Memory and Stress-Induced Alterations of Brain Structure and Function

**DOI:** 10.3390/ph3103143

**Published:** 2010-10-11

**Authors:** Phillip R. Zoladz, Carmen Muñoz, David M. Diamond

**Affiliations:** 1 Department of Psychology & Sociology, Ohio Northern University, Ada, OH 43222 USA; 2 Servier International, 35 rue de Verdun, 92284 Suresnes, France; 3 Research & Development Service, James A. Haley VA Hospital, Tampa, FL 33612, USA; 4 Department of Psychology, Cognitive and Neural Sciences Division, University of South Florida, Tampa, FL 33612, USA; 5 Department of Molecular Pharmacology and Physiology, University of South Florida, Tampa, FL 33612, USA

**Keywords:** antidepressant, synaptic plasticity, memory, animal model, glutamate, stress

## Abstract

Tianeptine is a well-described antidepressant which has been shown to prevent stress from producing deleterious effects on brain structure and function. Preclinical studies have shown that tianeptine blocks stress-induced alterations of neuronal morphology and synaptic plasticity. Moreover, tianeptine prevents stress from impairing learning and memory, and, importantly, demonstrates memory-enhancing properties in the absence of stress. Recent research has indicated that tianeptine works by normalizing glutamatergic neurotransmission, a mechanism of action that may underlie its effectiveness as an antidepressant. These findings emphasize the value in focusing on the mechanisms of action of tianeptine, and specifically, the glutamatergic system, in the development of novel pharmacotherapeutic strategies in the treatment of depression.

## 1. Introduction

Depression is a cause of significant distress and financial burden across the globe [1,2], and continued research assessing its etiology is essential to facilitate the development of better treatments for the disorder. Although considerable progress has been made in describing the physiological and behavioral *sequelae* that result from depression, the factors responsible for its development and maintenance remain largely unknown. A considerable proportion of what we know about the biological basis of this disorder has come about through studies examining pharmacological agents that treat it most effectively. Indeed, the dogmatic view that abnormally low levels of monoamine neurotransmitters result in depression developed out of the incidental finding that efficacious antidepressant agents, such as monoamine oxidase inhibitors and tricyclic antidepressants, substantially increased monoamine neurotransmitter levels [3]. Even today, the most frequently prescribed medications for depression are the selective serotonin reuptake inhibitors (SSRIs), whose primary mechanism of action involves increasing extracellular serotonin levels [4,5]. 

Recent work has suggested that the widely held view that depression results from abnormally low levels of monoamine neurotransmitters is an oversimplification of a much more complex process, and that elevated monoamine levels provides only an indirect contribution to therapy for depressive symptoms [6,7,8]. Thus, antidepressant agents with a primary mode of action to increase monoamine neurotransmitter levels, such as the SSRIs, are effective in only a subset of people suffering from depression [9,10,11]. Moreover, SSRIs are typically used in a polypharmacy (multi-drug) approach to the treatment of depression, which suggests that the focus on the SSRI-based serotonergic component of depression is incomplete. It is therefore evident that mechanisms other than alterations of monoamine neurotransmitter levels are involved in the development and maintenance of depression.

An alternative and well-established treatment for depression is tianeptine, a clinically effective antidepressant whose non-monoaminergic mechanism of action is quite unlike that of other pharmacological treatments for the disorder; tianeptine’s antidepressant effects primarily involve the modulation of glutamatergic neurotransmission and the modulation of the capacity for the brain to exhibit synaptic plasticity [9,12,13,14,15,16,17]. Tianeptine reduces depressive symptoms in individuals with mild to severe forms of the disorder, and unlike SSRI’s, tianeptine is effective with fewer side effects in a monotherapy approach [18,19,20,21,22]. Tianeptine’s effectiveness in treating depression is of clinical, as well as conceptual, significance. The contrast in mechanistic actions between tianeptine and other types of antidepressants serves as a challenge to the heuristic value of the monoamine hypothesis of the disorder [23,24]. 

In this review, we have provided an update on research on stress, depression and neuroplasticity, and more specifically, we have described the influence of tianeptine on cognitive and physiological measures of brain function. In addition, we have integrated the literature on stress, memory and synaptic plasticity with our recent work on the enhancement of long-term hippocampus-dependent memory by acute administration of tianeptine. These findings are potentially relevant toward the amelioration of cognitive deficits and hippocampal pathophysiology which are endemic to depression [25,26,27].

## 2. Stress, Neuroplasticity and Symptoms of Depression

Two commonalities to almost all mood and anxiety disorders include alterations in neuroplasticity and stress as contributing factors [28,29,30,31]. Alterations in neuroplasticity involve structural and functional changes in how the brain processes information. It has been hypothesized that many of the behavioral symptoms of depression are manifested through changes in brain neurochemical levels that ultimately result in structural changes in brain regions that process emotional and cognitive information, including the hippocampus, prefrontal cortex (PFC) and amygdala [16,32,33]. The hippocampus is a medial temporal lobe structure which is important for declarative memory in humans [34,35] and spatial working memory in rodents [36,37,38,39,40,41]; the PFC is located in the anterior part of the frontal lobe and plays an important role in complex cognitive processes, such as planning, decision-making and behavioral flexibility [42]; and, the amygdala is an almond-shaped structure in the medial temporal lobe which is highly involved in emotion and memory [43,44,45]. 

In support of the notion that depression involves alterations of neuroplasticity, studies have reported significant reductions of hippocampal and PFC volumes in depressed patients [46,47,48]. In addition, depressed individuals exhibit impaired performance on hippocampus- and PFC-dependent cognitive tasks, impairments that have been associated with reduced or abnormal activity in each of these respective brain regions while depressed patients were engaged in such tasks [49,50]. In contrast to the hippocampus and PFC, amygdala volume and activity are increased in depressed individuals, and with successful treatment, significantly decline [51,52,53]. Thus, depression is clearly associated with significant changes in brain structure and function, which may ultimately explain the expression of depression’s behavioral symptoms.

It has been well-established that stress is a significant contributor to one’s likelihood of developing depression [54,55]. Thus, a major focus of preclinical researchers has been to define the physiological and behavioral alterations that result from stress and to ascertain how they can be prevented through pharmacological means. Animal models of stress provide unique advantages over studying humans with depression in that they afford researchers a greater amount of control over experimental variables, and they allow the investigators to assess neurobiological endpoints (e.g., neuron structure, cellular and molecular measures) that would be difficult, if not impossible, to assess in human patients. The models are also useful because once they reveal the physiological and behavioral effects that stress exerts on brain structure and function, investigators can use this information to develop novel pharmacological agents in alleviating such effects. Of course, researchers must exert caution when applying the findings from animal models to the human disorder. Animal models are not necessarily capable of modeling all of the core symptoms of depression, and those symptoms that can be modeled do not always reflect the underlying mechanisms involved in the human situation [56]. Thus, animal models do provide researchers with several advantages in developing a better understanding of the neurobiological basis of depression, but the models must be employed and interpreted prudently.

Extensive preclinical research has shown that chronic stress produces physiological and behavioral alterations (e.g., abnormal hypothalamus-pituitary-adrenal (HPA) axis functioning, cellular and molecular abnormalities, anhedonia, learned helplessness, cognitive impairments) which are analogous to those observed in people with depression [57,58,59]. In terms of neuroplasticity, investigators have found that in animal models of chronic stress there is a significant reduction of the length, spine density and arborization of dendrites on neurons in the hippocampus [60,61,62,63,64,65,66,67] and PFC [68,69,70,71,72,73], and increases in each one of these parameters in neurons of the amygdala [66,74]. Correspondingly, these chronic stress regimens have been shown to produce significant impairments of hippocampus-dependent (e.g., spatial learning) [75,76,77,78,79,80,81] and PFC-dependent (e.g., attention set-shifting, reversal learning) [68,71] memory, while enhancing performance on amygdala-dependent tasks (e.g., fear conditioning) [60,82]. Moreover, the same chronic stress regimen that results in hypertrophy of amygdala neurons increases the expression of anxiety-like behaviors in rats tested on the elevated plus maze [66,74].

The effects of chronic stress on hippocampal [60,77] and PFC [72] morphology have been found to be reversible – that is, the dendrites of neurons in these brain regions re-grow when the chronic stress regimen is discontinued. On the other hand, the effects of chronic stress on amygdala morphology and the amygdala-mediated expression of anxiety-like behavior do not reverse following the termination of stress [83]. The ability of neurons in the hippocampus and PFC to recover after the termination of stress appears to depend on the age of the subject under investigation; a recent study revealed that young (*i.e.*, 3-month-old) rats exhibited full recovery of neurons in the PFC following cessation of chronic restraint stress, while middle-aged (*i.e.*, 12-month-old) and aged (*i.e.*, 20-month-old) rats exhibited minimal recovery in response to identical stress procedures [84]. This finding is particularly relevant to our understanding of how to develop better treatments for depression because it suggests that as the age of the individual increases, neuroplasticity, and the corresponding ability for neurons to recover following injuries that could be associated with depression, decreases. Therefore, if reversing the alterations of neuroplasticity that are induced by depression is key to successful treatment of the disorder, we must not look to develop pharmacological agents that merely prevent these alterations; rather, we should look to develop pharmacological agents that promote neuron growth and resilience.

Research over the past couple of decades has shown that the effects of chronic stress on the morphology and functionality of the hippocampus, PFC and amygdala are mediated by an interaction between glucocorticoids and *N*-methyl-D-aspartate (NMDA) receptor activity. For instance, chronic administration of corticosterone mimics the effects of chronic stress on hippocampal [77,85,86] and PFC [87] morphology, and the stress-induced dendritic retraction observed in the hippocampus is blocked by steroid synthesis inhibitors [88], as well as NMDA receptor antagonists [88] and agents that significantly reduce extracellular levels of glutamate (e.g., phenytoin) [64,89]. These findings resonate with research in depressed patients, which indicates that these individuals exhibit an overactive HPA axis [90,91] and abnormal brain glutamatergic levels [92,93,94].

## 3. Tianeptine Prevents Stress-Induced Alterations of Neuroplasticity

Daily administration of tianeptine has been shown to prevent the chronic stress-induced reduction of overall hippocampal volume [95] and CA3 dendrites [60,96,97], as well as the chronic stress-induced hypertrophy of neurons in the amygdala [16,98]. Additional work has revealed that tianeptine prevents the effects of chronic stress on hippocampus-dependent learning and memory [79,99,100] and the amygdala-mediated enhancement of anxiety-like behavior [16,98]. The latter finding may be relevant to other work reporting that chronic tianeptine treatment reduces the expression of auditory fear conditioning, an amygdala-dependent task [101]. It is notable that the SSRIs fluoxetine and fluvoxamine were ineffective in preventing the effects of chronic stress on CA3 morphology [96], providing compelling evidence that SSRIs and tianeptine act through different cellular and molecular mechanisms.

The hippocampus is one of only two brain regions in the adult mammalian brain that produces new neurons through a process known as neurogenesis [102]. Although the functional role of neurogenesis has remained a highly debated topic, studies have provided evidence linking hippocampal neurogenesis with hippocampus-dependent learning and memory [103,104]. In addition, researchers have hypothesized that the pathogenesis of depression involves impaired hippocampal neurogenesis [105,106,107,108,109]. Accordingly, in animal models, chronic stress significantly reduces hippocampal neurogenesis [110,111,112,113,114,115] and increases apoptotic cell death in the hippocampus and temporal cortex [111,116,117]. Clinically effective antidepressants, including tianeptine, prevent the effects of chronic stress on hippocampal neurogenesis [95,105,115]. Tianeptine has also been reported to block the chronic stress-induced increase in apoptotic cell death in the temporal cortex [118], which may be related to its prevention of the chronic stress-induced reduction of cerebral metabolites associated with neuronal viability (e.g., *N*-acetyl-aspartate) [95].

Neurotrophic factors are significant regulators of cell survival and proliferation, thus making them vitally important for the process of neurogenesis [119]. Some of the most extensively characterized neurotrophic factors include nerve growth factor (NGF), brain-derived neurotrophic factor (BDNF), neurotrophin-3 (NT-3) and neurotrophin-4 (NT-4). Numerous studies have shown that acute and chronic stress significantly reduce neurotrophic factor levels [115,120,121,122], with many of the studies focusing on the stress-induced reduction of BDNF levels in the hippocampus [114,123,124,125,126,127,128,129,130,131]. This effect has become the center of attention, at least in part, because several studies have reported significantly reduced levels of serum and hippocampal BDNF in depressed patients [132,133,134]. BDNF knock-out mice have been reported to exhibit morphological changes in the hippocampus that are comparable to those observed following exposure to chronic restraint stress [135]. Interestingly, investigators have shown that the efficacy of antidepressants in ameliorating behavioral symptoms of depression in depressed patients and in animal models of stress depends on their ability to increase BDNF levels [134,136,137].

Tianeptine’s prevention of the effects of chronic stress on neurogenesis may involve blocking the stress-induced reduction of neurotrophic factor levels in the hippocampus [123]. Another study found that chronic tianeptine treatment significantly increased BDNF levels in the rat amygdala, independent of whether or not the rats were exposed to stress [135]. According to Reagan and colleagues, the amygdala may be the site of the initiation of chronic stress-induced morphological changes in other brain regions, such as the hippocampus and PFC [135,138]. In support of this hypothesis, clinical studies on depressed patients have reported that morphological changes in the amygdala precede those that are observed in the hippocampus [139]. Therefore, tianeptine’s effectiveness as an antidepressant treatment may result from its enhancement of neurotrophin actions in the amygdala. 

## 4. Stress and Synaptic Plasticity: Stabilization by Tianeptine

Synaptic plasticity involves activity-induced changes in synaptic function which then affect how the synapse will subsequently respond to afferent activity. Synaptic plasticity has long been hypothesized to be important for learning and memory, and it has been speculated that people with depression exhibit abnormal synaptic plasticity in the hippocampus, PFC and amygdala [16,17,30]. To indirectly address this issue, investigators have used animal models to examine the effects of stress on long-term potentiation (LTP), a physiological model of learning and memory involving an enhancement of synaptic efficacy following high-frequency stimulation of afferent fibers [140]. Extensive work has shown that stress impairs the induction of LTP in the hippocampus and PFC, while facilitating its induction in the amygdala [141,142,143,144]. This stress-induced modulation of synaptic plasticity has been shown to be mediated by interactions among glucocorticoids [145,146,147], glutamatergic NMDA receptors [148,149,150] and amygdala-induced modulation of hippocampal plasticity [151,152].

Tianeptine has been shown to block the stress-induced impairment of LTP in the hippocampus and PFC, without interfering with the stress-induced enhancement of LTP in the basolateral amygdala (BLA) [153,154,155,156]. Tianeptine blocked the inhibitory effects of stress on hippocampal LTP and primed burst potentiation (PBP), a low-threshold form of LTP, when it was administered either before or after the stress experience [155,156]. Other antidepressants, including some SSRIs, have also been reported to block the effects of stress on LTP in the hippocampus and PFC, although these effects have been less significant and more transitory in nature [154,157].

## 5. Tianeptine Protects Memory from Stress and Enhances Learning and Memory

Extensive work has shown that acute stress impairs hippocampus-dependent learning and memory in humans and rodents [141,142,143,144,158,159]. We previously reported that tianeptine, but not the anxiolytic propranolol, blocked the predator stress-induced impairment of rat spatial memory in the radial-arm water maze (RAWM) [160]. The RAWM is a water-filled tank with six swim arms radiating from an open central area; a hidden platform is placed at the end of one of the arms, and rats are given several training trials to learn the location of the hidden platform. Tianeptine prevented the effects of stress on memory without altering the stress-induced increase in glucocorticoids, indicating that tianeptine’s memory-protective effects can occur without attenuating the stress-induced activation of the HPA axis. This finding was also consistent with *in vivo* electrophysiological studies reporting that tianeptine blocked the effects of stress on hippocampal LTP without affecting stress-induced increases in corticosterone levels in rats [155]. 

In more recent work, we reported that predator stress impaired spatial memory in rats that were adrenalectomized (ADX) [161]. This finding demonstrates that exposure to a cat can impair memory in the absence of a stress-induced increase in glucocorticoid levels. This finding, alone, provides strong evidence that the acute stress-induced impairment of hippocampus-dependent learning does not require a stress-induced elevation of glucocorticoid levels. More importantly, we found that tianeptine prevented the stress-induced impairment of spatial learning in ADX animals, as well. Collectively, these findings provide convincing evidence that, under stress conditions, tianeptine’s memory-protective effects are not accomplished by modulation of glucocorticoid levels. 

Studies have also shown that tianeptine administration, under non-stress conditions, increases the magnitude of synaptic plasticity (LTP and PB potentiation) in the hippocampal CA1 region [155,156]. This finding suggests that tianeptine should enhance learning and memory. Indeed, studies have shown that tianeptine enhances spontaneous alternation behavior, as well as performance on discrimination tasks in the T-maze and radial arm maze [162,163]. In contrast, acute administration of the SSRI fluoxetine impaired performance on the radial arm maze discrimination task [162], a finding that is relevant to other work reporting that fluoxetine impairs the induction of LTP in hippocampal slices [155].

We have extended the findings on tianeptine’s procognitive effects by assessing its influence on the spatial memory of rats trained in the RAWM. In the experiment, rats (250–275 g; Charles River Laboratories) were injected intraperitoneally with one of five doses of tianeptine (0.25, 0.50, 1, 5, 10 mg/kg) or vehicle (0.9% saline, 1 mL/kg) and then 30 min later, the rats were given RAWM training, following previously-described methods [78,160,161,164,165,166,167,168,169,170,171]. Briefly, the rats were given 4 trials to learn the location of a hidden escape platform, which was placed at the end of one of six arms, in the RAWM. Arm entry errors (*i.e.*, entries into arms that did not contain the hidden platform) served as an indicator of the rat’s accuracy of its memory for the hidden platform location. Following the 4 acquisition trials, the rats spent a 1-hour delay period in their home cages. This delay period terminated with a single short-term memory test trial in the RAWM. Twenty-four hours later, the rats were given a single memory test trial in the RAWM to assess their long-term memory for the hidden platform location. The doses of tianeptine used in this experiment include the same doses that have been shown to enhance hippocampal LTP and PBP [16,17,155,156], and to block the effects of chronic stress on hippocampal morphology [60,95,96,97] and hippocampus-dependent learning and memory [79,99,100].

**Figure 1:**
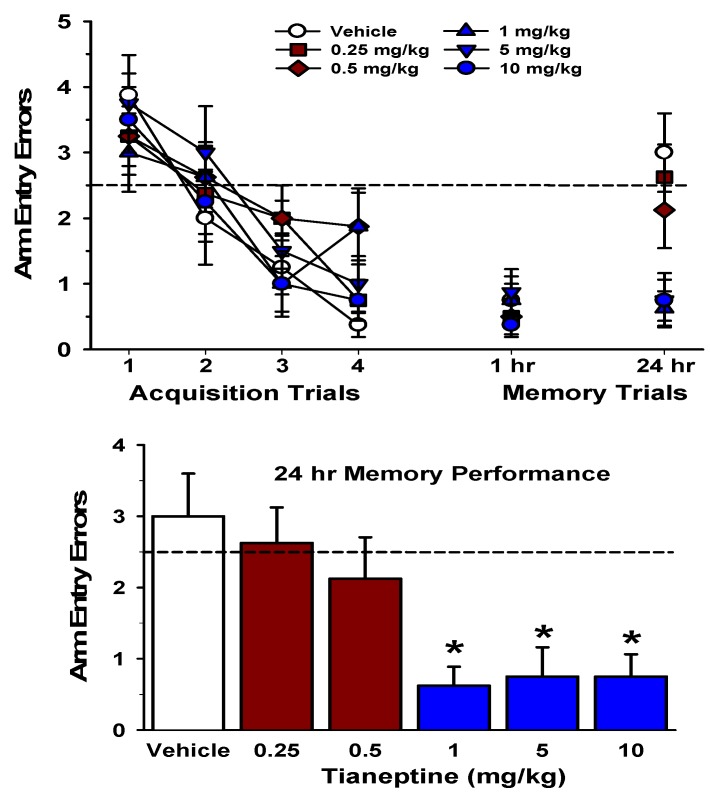
Pre-training administration of tianeptine enhanced long-term (24-hours) spatial memory in the RAWM.

The top graph illustrates arm entry errors during the four acquisition trials in the RAWM (1–4; left) and memory test trial performance 1 and 24 hours later. The acquisition data were analyzed with a mixed-model ANOVA, which revealed a significant main effect of trials, *F* (3,126) = 25.67, *p* <0.001, indicating that the rats made significantly fewer errors as the trials progressed. There was no significant main effect of group, and the Trial x Group interaction was not significant (*p*’s >0.05). Arm entry errors from the 1-hour and 24-hours memory test trials were analyzed with separated one-way ANOVAs. For the 1-hour memory test trial, there was no significant group effect (*p* >0.05). The 24-hours memory test trial is shown at the right of the top graph and also in the lower graph. There was a significant group effect for the 24-hours memory performance data, *F* (5,42) = 5.32, *p* <0.001. Post hoc tests indicated that the groups administered 1, 5, or 10 mg/kg of tianeptine made significantly fewer arm entry errors than the groups administered vehicle (data in blue). The lower graph illustrates 24-hours memory performance in greater detail. The tianeptine-treated groups which exhibited no significant improvement in memory (0.25 and 0.5 mg/kg) are represented by the brown bars, and the tianeptine-treated groups which exhibited a significant improvement in memory (1, 5 and 10 mg/kg) are represented by the blue bars. The dashed line at 2.5 errors in both graphs indicates chance level of performance [168]. * = *p* <0.05 relative to the vehicle-treated group.

As illustrated in Figure 1 (top), the tianeptine- and vehicle-treated groups were equivalent in their performance during the acquisition phase of the water maze task, as all groups made significantly fewer arm entry errors across the 4 training (acquisition) trials. In addition, all groups exhibited statistically equivalent performance on the short-term (1-hour) spatial memory test trial. The specific effect of pre-training tianeptine administration was revealed on the 24-hours memory test. Rats injected with the vehicle or the lowest doses of tianeptine (0.25 or 0.5 mg/kg) exhibited a deterioration of their memory at 24-hours. Rats administered the higher doses of tianeptine (1, 5, or 10 mg/kg), in contrast, exhibited intact 24-hours memory. This finding indicates that tianeptine, when administered 30 minutes prior to acquisition, produced a dose-dependent enhancement of long-term (24-hours) spatial memory in rats. 

The tianeptine-mediated enhancement long-term memory is likely to be based on tianeptine’s well-described enhancement of physiological measures of hippocampal function, including an enhancement of synaptic plasticity in the CA1 region. Moreover, these findings are consistent with our previous speculation that tianeptine enables hippocampus-dependent information to be stored more efficiently, thereby enhancing long-term memory under control conditions (Figure 1), as well as to protect the retrieval of the memory from being disrupted by stress [160].

## 6. Mechanisms Underlying Tianeptine’s Procognitive and Anti-Stress Effects

Recent studies have indicated that tianeptine’s therapeutic effects are associated with its modulation of the glutamatergic system [7,8,12,16,33,161,172]. Glutamate is the primary excitatory neurotransmitter of the central nervous system, and one of its roles is to regulate calcium influx by acting on postsynaptic AMPA and NMDA receptors [173]. Studies have shown that depressed patients exhibit elevated glutamate levels in plasma, CSF and post-mortem brain samples, which supports current views implicating the dysregulation of glutamate transmission in the pathogenesis of depression [92,93,94]. 

Extensive work has implicated hyperactivity of the glutamatergic system in the deleterious effects of stress on brain structure and function. Experiments conducted primarily on the hippocampus have shown that stress significantly increases glutamate levels [138,174,175,176,177], inhibits glutamate uptake [178], increases the expression and binding of glutamate receptors [179,180,181] and increases calcium currents [182]. Accordingly, researchers have shown that administration of NMDA receptor antagonists blocks the effects of stress on behavioral, morphological and electrophysiological measures of hippocampal function [148].

Tianeptine appears to protect the hippocampus and PFC from the deleterious effects of stress and depression by normalizing the stress-induced modulation of glutamatergic activity. For instance, tianeptine blocked the stress-induced increase in NMDA channel currents, as well as the ratio of NMDA: non-NMDA receptor currents, in the hippocampus [183]. Tianeptine also inhibited the acute stress-induced increase in extracellular levels of glutamate in the BLA, while having no effect on the stress-induced increase in these levels in the central nuclei of the amygdala (CeA) [138]. Interestingly, as mentioned above, tianeptine had no effect on the stress-induced enhancement of LTP in the BLA [156]. This finding suggests that the stress-induced enhancement of LTP in the BLA may involve NMDA-independent forms of synaptic plasticity, such as voltage-gated calcium channel-dependent LTP [184]. 

In contrast to tianeptine, administration of the SSRI fluoxetine increased baseline and stress-induced levels of glutamate in the BLA and CeA [138]. This finding may explain why SSRIs are anxiogenic early in the treatment phase [101,185]. Moreover, investigators have shown that acute administration of the SSRI citalopram enhanced the acquisition of auditory fear conditioning, while chronic treatment with citalopram impaired the acquisition and expression of conditioned fear [101]. Acute treatment with tianeptine, in contrast, had no effect on auditory fear conditioning, but when given chronically, exerted effects comparable to those of citalopram. Thus, tianeptine demonstrates long-lasting anxiolytic and antidepressant effects that are similar to SSRIs, without the adverse acute effects typically found with these agents.

Tianeptine’s effect on glutamatergic activity in the amygdala may play an important role in its ability to reverse the effects of chronic stress on amygdala morphology and the expression of anxiety-like behaviors. In addition to its glutamatergic modulation, tianeptine reduces the expression of corticotropin-releasing hormone (CRH) mRNA in the amygdala and the bed nucleus of the stria terminalis (BNST), a brain region that is highly innervated by amygdala fibers [186]. CRH neurotransmission in both of these regions has been implicated in the expression of anxiety-like behaviors, and several studies have reported significantly elevated CSF CRH levels in depressed patients [187,188,189]. If the amygdala is the site of the initiation of chronic stress-induced functional changes in other brain regions, such as the hippocampus and PFC, then tianeptine’s ability to stabilize amygdala activity could underlie its widespread anti-stress effects.

Chronic stress has been shown to increase expression of the glutamate transporter, GLT-1, which is important for removing excess glutamate from synaptic regions [190]. This effect was specifically observed in the CA3 region of the hippocampus, the primary area exhibiting significant morphological alterations following chronic stress. Researchers have postulated that the up-regulation of GLT-1 levels in this region is a compensatory response to chronic elevations of extracellular glutamate levels. Importantly, tianeptine has been shown to block the stress-induced increase in hippocampal GLT-1 levels. In theory, tianeptine accomplishes this feat by normalizing stress-induced glutamate levels in the hippocampus, thereby removing the stimulus (*i.e.*, excessive glutamate) which necessitates increased expression of GLT-1.

In addition to its ability to normalize the stress-induced increase in NMDA receptor currents, tianeptine also increases basal excitatory synaptic transmission in hippocampal circuits, predominantly via enhancing AMPA EPSCs [183]. In addition to NMDA receptors, AMPA receptors play an important role in excitatory synaptic transmission and the induction of long-term synaptic plasticity [191]. Recent work has reported that tianeptine modulates the phosphorylation of AMPA receptor subunits in the hippocampus [6]. Other antidepressants, such as SSRIs and tricyclic antidepressants, have been shown to increase phosphorylation of the Ser845 site on the glutamate receptor subunit 1 (GluR1) of hippocampal AMPA receptors [192,193]. One team of investigators found that chronic, but not acute, tianeptine treatment significantly increased phosphorylation of the Ser831 and Ser845 sites on the GluR1 subunit of AMPA receptors in the CA3 region of the hippocampus [6]. More recent work has shown that acute tianeptine significantly increased phosphorylation of the Ser831 site on the GluR1 subunit of AMPA receptors in the PFC, which was associated with tianeptine’s ability to prevent the acute stress-induced impairment of LTP in the hippocampus-PFC pathway [157]. Typically, phosphorylation of the Ser831 and Ser845 sites of AMPA receptors occurs via protein kinase A (PKA) and calcium/calmodulin-dependent protein kinase II (CaMKII) or protein kinase C (PKC), respectively, and potentiates AMPA currents in the hippocampus [194,195]. Thus, the tianeptine-mediated increase in the phosphorylation of the serine sites on the GluR1 subunit of AMPA receptors could explain the finding of a tianeptine-induced enhancement of AMPA EPSCs in the study of Kole *et al*. [183], which may also be relevant toward understanding tianeptine’s effectiveness as an antidepressant.

In recent work, Uzbay and colleagues found that tianeptine attenuated pentylenetetrazole (PTZ)-induced seizures [196,197,198] in rodents, which is consistent with its known mode of action to reduce excessive glutamatergic activity. Importantly, the investigators found that the latter effect was blocked by the administration of caffeine, a nonspecific adenosine receptor antagonist, and 8-cyclopentyl-1,3-dipropylxanthine, an A_1_ receptor-specific antagonist. Administration of the A_2_ receptor-specific antagonist, 8-(3-chlorostyryl) caffeine, had no effect on the tianeptine-induced delay of seizure onset. Thus, tianeptine’s anticonvulsant and anxiolytic properties in rodents [98,101,199,200,201,202,203,204] and in depressed people [205,206] may involve stabilization of glutamate levels acting in concert with activation of A_1_ adenosine receptors [199,200,201,202].

## 7. Summary and Conclusions

Considerable progress has been made in describing the physiological and behavioral sequelae that result from depression, but the specific factors responsible for its development and maintenance are not well understood. Investigators have utilized animal models of stress effects on brain and behavior to develop a better understanding of the neurobiological basis of depression, which could ultimately produce improved treatment options for the patient. We have reviewed the findings of preclinical research demonstrating that tianeptine prevents the deleterious effects of stress on physiology and behavior. Tianeptine prevents chronic stress-induced morphological changes in the hippocampus and amygdala and blocks the effects of acute stress on synaptic plasticity in the hippocampus and PFC. We have also reviewed findings demonstrating that tianeptine has procognitive effects. Tianeptine enhances hippocampus-dependent learning and memory and prevents the stress-induced impairment of such processes. Tianeptine’s prevention of the adverse effects of stress on brain and behavior is likely to contribute to its effectiveness as a treatment for people suffering from depression. 

Tianeptine’s antidepressant effects appear to involve modulation of glutamatergic neurotransmission, which resonates with evidence implicating abnormal glutamate activity in the pathogenesis of depression. Cellular, molecular and electrophysiological studies have shown that tianeptine prevents the stress-induced rise in amygdaloid glutamate levels and blocks stress-induced changes in glutamate receptor currents and glutamate transporter expression in the hippocampus. Moreover, tianeptine potentiates AMPA receptor function, as demonstrated by increasing phosphorylation of the Ser831 and Ser845 sites on the GluR1 subunit of AMPA receptors in the hippocampus and PFC. These latter findings may explain why tianeptine enhanced long-term (24-hours) hippocampus-dependent memory retrieval (as reported here) and, more generally, how it facilitates synaptic plasticity in the hippocampus. Other research has shown that tianeptine has anticonvulsant properties, which appear to be based on its stabilization of glutamate levels in conjunction with adenosine receptor activation. 

In summary, tianeptine is a well-described antidepressant with effective actions against stress-induced deficits of the nervous system. It is as effective as SSRIs in treating depression, produces fewer adverse side effects and reduces anxious symptoms associated with depression without the need for concomitant anxiolytic therapy [18,19,20,21,207]. It is therefore relevant to note that tianeptine has been shown to ameliorate symptoms in people with post-traumatic stress disorder (PTSD) [208] and in recent work has been shown to block the effects of intense stress on behavior and cardiovascular systems in an animal model of PTSD [100]. Thus, the well-described antidepressant and memory protective properties of tianeptine indicate that, in addition to its effectiveness as a treatment in mood disorders, it potentially has broader applications, as in the treatment of anxiety.
